# The effectiveness of an online interdisciplinary intervention for mental health promotion: a randomized controlled trial

**DOI:** 10.1186/s40359-021-00577-8

**Published:** 2021-05-11

**Authors:** Geraldine Przybylko, Darren Morton, Lillian Kent, Jason Morton, Jason Hinze, Peter Beamish, Mel Renfrew

**Affiliations:** 1 Lifestyle Medicine and Health Research Centre, Avondale University College, 582 Freemans Drive, Cooranbong, NSW 2265 Australia; 2 Faculty of Education, Business and Science, Avondale University College, 582 Freemans Drive, Cooranbong, NSW 2265 Australia

**Keywords:** Mental health promotion, Lifestyle medicine, Positive psychology, Randomized controlled trial, Online, Interdisciplinary

## Abstract

**Background:**

There is an urgent need for efficacious interventions to combat the global mental health crisis, and mental health promotion and primary prevention approaches are paramount. The aim of this study is to examine whether an online interdisciplinary intervention that incorporates evidence-based strategies from the disciplines of Lifestyle Medicine and Positive Psychology improves measures of mental health and emotional wellness.

**Methods:**

A randomized controlled trial with a wait-list control (N = 425, aged 46.97 ± 14.5, 69.9% females) was conducted in Australia and New Zealand. The intervention group participated in a 10-week online interdisciplinary intervention. Primary outcome measures of mental health and emotional wellness were taken at baseline (Week 1), post-intervention (Week 12), and 12 weeks post-intervention (Week 24). The wait-list control completed the same assessments.

**Results:**

General Linear Modelling analyses indicated that the intervention group experienced significantly greater improvements than the wait-list control group over time in all outcome measures: mental health (F(319) = 7.326, *p* = 0.007) and vitality (F(319) = 9.445, *p* = 0.002) subscales of the Short Form Survey (SF-36); depression (F(319) = 7.841, *p* = 0.005), anxiety (F(319) = 4.440, *p* = 0.36) and stress (F(319) = 12.494, *p* < 0.001) scales of the Depression, Anxiety and Stress Scale (DASS-21); and life satisfaction (F(319) = 8.731, *p* = 0.003) as measured by the Satisfaction With Life Scale. Within the intervention group, significant improvements were observed from Week 1 to 12 in all outcome measures: mental health (10%, t(167) = − 6.423), *p* < 0.001, dz = 0.50), vitality (22%, t(167) = − 7.043, *p* < 0.001, dz = 0.54), depression (− 41%, t(167) = 6.189, *p* < 0.001, dz = 0.48), anxiety (− 38%, t(167) = 5.030, *p* < 0.001, dz = 0.39), stress (− 31%, t(167) = 6.702, *p* < 0.001, dz = 0.52) and life satisfaction (8%, t(167) = − 6.199, *p* < 0.001, dz = 0.48). Improvements in the outcome measures remained significant in the intervention group at 12 weeks post-intervention.

**Conclusion:**

The online interdisciplinary intervention improved measures of mental health and emotional wellness suggesting that such interventions may be useful for mental health promotion and prevention.

*Trial registration* The Australian New Zealand Clinical Trials Registry. ACTRN12619000993190. Registered on 12 July 2019 (Retrospectively registered). The ANZCTRN is part of the WHO Primary Registries.

**Supplementary Information:**

The online version contains supplementary material available at 10.1186/s40359-021-00577-8.

## Background

Mental health disorders have reached epidemic proportions worldwide [1]. In the United States (U.S.), the burgeoning costs of mental disorders constituent the most costly medical condition, amounting to 201 billion U.S. dollars annually, which surpasses heart conditions (147 billion U.S. dollars), trauma (143 billion U.S. dollars) and cancer (122 billion U.S. dollars) [2]. The current paradigm for the frontline treatment of affective disorders centres on pharmacological intervention. Antidepressant usage has doubled over the past decade in the United Kingdom [3]; is now ranked in the top three most commonly used therapeutic drug classes in the U.S. [4]; and is the most commonly used psychotropic medication in Australia [5]. Despite this increase, the incidence of depression continues to escalate [5–7].

This has led to repeated calls to address mental health on a population-level using a more integrative approach that includes non-pharmacological strategies such as lifestyle interventions, mental health promotion, education programs and psychological therapies [5, 7–12]. Greater focus on mental health promotion and prevention initiatives are needed to enhance the mental health on a population-level to serve as a protective buffer against mental illness [13] and reduce the burden of mental health disorders [14].

Over the past few decades, numerous evidence-based strategies for improving mental health and emotional wellness have emerged in the literature underpinning the disciplines of ‘Lifestyle Medicine’ and ‘Positive Psychology’ [15–18]. Lifestyle Medicine has historically focused on the prevention, management and reversal of chronic diseases through the promotion of exercise, a healthy diet, and sleep [16, 19–26], however, there is growing evidence that these lifestyle practices also have positive benefits on mental health [9, 27–31], For example, considerable literature is showing that dietary interventions can be used as an effective treatment strategy for depression [32]. Further, a meta-analysis has shown that exercise is beneficial for mental health [33]. The causal relationship between insomnia and depression [34] is well established; and quality sleep is known to be paramount to good physical and mental health [35, 36].

Positive Psychology focuses on pathways to promote human flourishing through exercises such as practicing gratitude, activating signature strengths, engaging in service activities and nurturing relationships [37–40]. The conclusion of two meta-analyses was that Positive Psychology interventions significantly enhance emotional wellbeing and decrease depressive symptoms [14, 41].

Lifestyle Medicine and Positive Psychology strategies have demonstrated efficacy for enhancing mental health and emotional wellness, however, they are often used in isolation. Interestingly, practitioners have been encouraged to prescribe multiple Positive Psychology strategies in a “shotgun approach”, rather than using a single strategy, as it may be more efficacious for their clients [41]. Additionally, the potential “compounding effects” of combining diet and exercise together, warrants further investigation of a more integrated approach using multiple lifestyle modifications [32].

Advances in online technology presents an opportunity to provide mental health promotion and primary prevention strategies that are easily accessible and available population-wide,  as well as overcome barriers with face-to-face interventions and the stigma of mental health disorders [42–45]. Online interventions can also provide low-cost solutions for dissemination on a population-level.

This study investigated the effectiveness of a 10-week online interdisciplinary intervention, incorporating both Lifestyle Medicine and Positive Psychology strategies, for improving the mental health and emotional wellness of a community-based cohort.

## Methods

### Design and participants

**Fig. 1:**
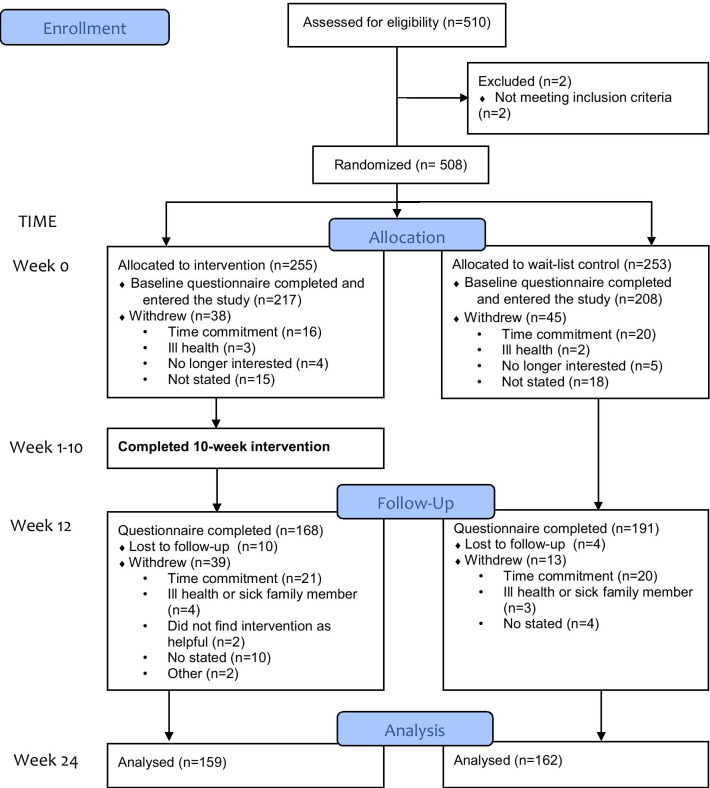
The Live More Project Intervention CONSORT diagram

The study used a non-blinded randomized controlled design. The treatment group participated in a 10-week intervention consisting of themed weekly sessions (see Table 1). Measurements of mental health and emotional wellness (mental health, vitality, depression, anxiety, stress and life satisfaction) were taken at baseline (Week 1), post-intervention (Week 12) and 12 weeks post-intervention (Week 24) (see Fig. 1). The wait-list control underwent the same measurements at times corresponding to the intervention group. The intervention was made freely available to the wait-list control at the completion of the study. Data was collected from July 2017 to February 2018 and analysed between 2018 and 2020. All procedures involving human subjects were approved by Avondale Human Research Ethics Committee [project number 2017:13]. The trial protocol is registered at The Australian New Zealand Clinical Trials Registry (ACTRN12619000993190).

**Table 1:** Weekly topics and challenges for the Live More Project Intervention

Week/topic	Daily challenge	Weekly challenge
1. Language and Emotion	Offer a genuine compliment	Memorise an inspirational text or saying
2. Posture and Regular Physical Activity	Spend 30 min of moderate exercise or 10,000 steps	20 min of guided resistance exercises
3. Sunlight and Natural Environments	Spend 30 min in an uplifting natural environment	Experience a sunrise
4. Social Connections	Do something intentional to show you care	Forgive someone who has hurt you
5. Positive Outlook	Spend 15 min to reflect on three things that went well	Write a letter of gratitude to someone and share it with them
6. Diet and the Gut Health Connection	Eat eight serves of plant-based food	Prepare a high-fibre, plant-based meal with one or more friends
7. Rest	Spend eight hours in bed without a device	Spend an evening by firelight
8. Stress Management	Spend 15 min in a quiet place, relaxing and being mindful of surroundings	Take a day off work and a digital Sabbath (going “off-line” for 24 hr to recharge)
9. Signature Strengths and Serving Smart	Perform a random act of kindness	Use signature strength to perform an act of service
10. Flourishing	Continue challenges found to be helpful	Continue challenges found to be helpful

The participants comprised of self-selected men and women from Australia and New Zealand who were recruited through a faith-based organization. The study was advertised in the faith-based organization’s internal communication channels including bulletins and magazines. The advertising materials did not target a clinical population. Instead, the intervention was promoted as an “emotional wellness” program. Inclusion criteria for participation in the study included: 18 years and older; fluent in English; and email and internet access. The inability to participate in moderate-intensity physical activity, such as walking, was an exclusion criteria for the study. Before randomization, the subjects agreed to participate in either arm of the study. The participants were then randomized into the intervention or wait-list control by a non-member of the research team using computer random number generation. Participants were then notified of their group allocation and were required to complete their enrollment and submit their informed consent.

### Intervention

The intervention, referred to as “The Live More Project” or “The Lift Project” [46–49], is a 10-week program that integrates strategies from the Lifestyle Medicine and Positive Psychology literature. Underpinning the intervention is the Theory of Planned Behaviour (TPB) that aimed to facilitate behavioral change through three key areas: a shift in attitude towards emotional wellness through education; a change in perceived norms by promoting social engagement; and an increase in perceived control by encouraging the participants to achieve weekly challenges [50].

The intervention used an experiential pedagogical framework of Learn, Experience, Think, and Share (LETS) [46] facilitated through an e-learning management system. In each weekly session, the participants viewed a themed educational video that presented evidence-based strategies for promoting mental health and emotional wellness. The participants were then encouraged to engage in daily and weekly challenges by practically applying the lessons learnt from each topic (Table 1). The participants were awarded challenge points for successfully completing the tasks with a maximum of 100 challenge points per week. The e-learning management system incorporated gamification by ranking the participants’ challenge points on a leaderboard. The e-learning management system also included a social forum that allowed interaction between the participants. The participants were able to post pictures and comments relating to the challenges. The participants also received reading materials to expand upon the content presented in the videos, an e-workbook for journaling and reminder notifications to complete the weekly topics and challenges.

### Outcomes and measures

All participants completed a self-reported wellness questionnaire, called the ‘7 Dimensions of Wellness Index’, three times during the study corresponding to baseline (Week 1), post-intervention (Week 12), and 12 weeks post-intervention (Week 24) of the intervention group. The questionnaire surveyed sociodemographic characteristics including age, sex ethnicity, marriage status, level of education, country of birth and validated instruments to determine the primary outcomes of mental health and emotional wellness as, outlined below.

#### Mental Health and Vitality

The 36-item Short Form Health Survey is deemed appropriate for use in general populations [51]. Two subscales where used in this present study: mental health (5 items) and vitality (4 items) subscale [52]. Studies have shown a Cronbach alpha of 0.90 for the mental health subscale and 0.87 for the vitality scale, indicating a good internal consistency [53, 54]. The present study observed a Cronbach alpha of 0.88 for mental health, 0.85 for vitality and 0.90 overall, indicating a good internal consistency.

#### Depression, Anxiety and Stress Scale

The 21-item Depression, Anxiety and Stress Scale (DASS-21) is suitable to measure depression, anxiety and stress (7 items per subscale) in both clinical and nonclinical populations [55]. Studies have indicated a good internal consistency with a Cronbach alpha range of 0.76 to 0.91 for the 3 subscales [56, 57] and > 0.90 for an overall score [58]. The present study observed a good internal consistency with a Cronbach alpha of 0.86 for depression, 0.68 for anxiety, 0.82 for stress and 0.79 for an overall score.

#### Satisfaction With Life Scale

The 5-item Satisfaction With Life Scale (SWLS) is used to measure global life satisfaction [59] across a broad age-range and applications [60]. A meta-analysis observed good internal consistency with a mean Cronbach alpha of 0.78 [61]. The present study observed a Cronbach alpha of 0.88 indicating good internal consistency.

### Sample size calculation

The sample size was calculated using assumptions based on published pilot data [47] and included the following assumptions: equal allocation of participants to each group; an improvement in depression, anxiety and stress scores of over 20% within the treatment group; a 40% attrition rate, based on reported levels of attrition in other online interventions [39]; 80% power and significance level of 0.05 (95% confidence interval). A small to moderate effect size was predicted based on comparative primary prevention interventions [62].

### Statistical analysis

The data were analysed using SPSS Statistics (version 25). Descriptive statistics, involving frequencies, means, standard deviations and confidence intervals were used to present the data. Repeated-measures analysis of variance (ANOVA), using the General Linear Modelling (GLM) function in SPSS, was used to test for group effects (intervention versus control), time effects (baseline to Week 12 and Week 24) and group versus time interactions. Only data from participants who had completed all three assessment periods were included in the analyses, except where specified. When significant, Bonferroni post-hoc analyses were used to determine significant changes from baseline to Week 12 and Week 24. Pearson’s correlation analysis was used to report the relationship between the outcome measures (see Additional file 2). The standardized difference method was used to calculate effect sizes (Cohen’s dz), calculated as the mean change between the respective timepoints divided by the standard deviation of the subjects’ mean difference scores. Missing data for age (n = 4) was replaced with the mean age and missing data for mental health outcomes were not included in the analysis.

## Results

**Fig. 2:**
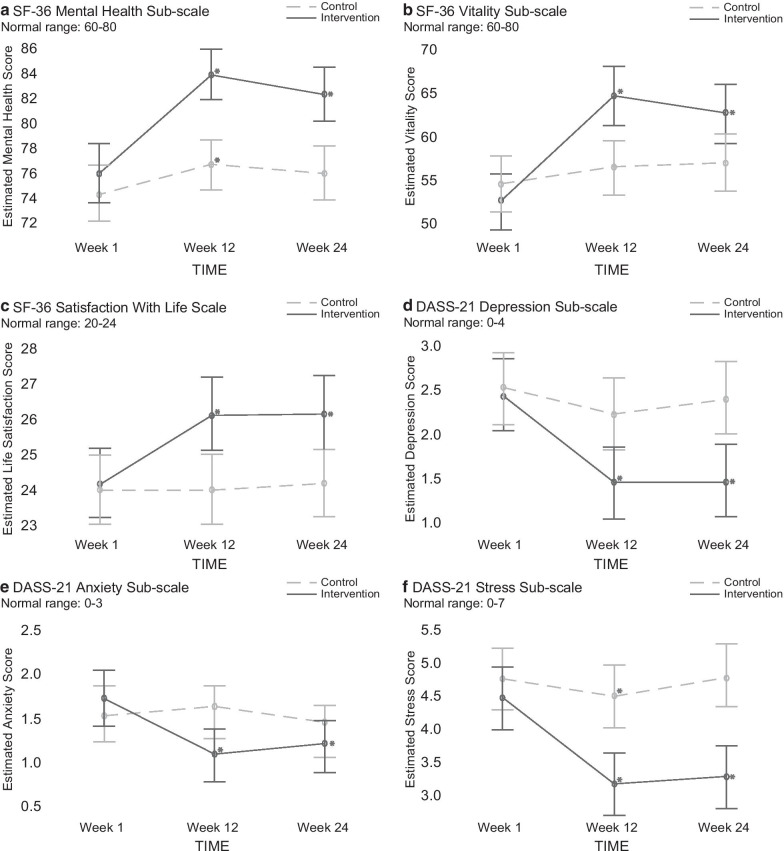
Mean scores on the outcomes measured using the SF-36 and SWLS (positive affect) and DASS-21 (negative affect) scales across time (Week 1, Week 12 and Week 24), showing 95% confidence levels. * Indicates a significant difference compared to Week 1

Between July and August 2017, 510 participants self-selected to participate in the study (see Fig. 1). The trial commenced in September 2017 and was completed February 2018 (Week 24). Of the 508 eligible participants, 425 (69.9% females, aged 46.97 ± 14.50) completed the baseline assessment and entered the study (217 intervention, 208 control). A total of 359 (85%) participants completed the post-intervention questionnaire (168 intervention, 191 control participants) and 321 (76%) completed the 12 weeks post-intervention questionnaire (159 intervention, 162 control). As shown in Table 2, the intervention and wait-list control were similar in age, sex, ethnicity, and education for the analysed data. There were no significant differences between the intervention and wait-list control in the baseline measures of: mental health (t(320) = − 0.843, *p* = 0.400) and vitality (t(320) = 0.881, *p* = 0.379) subscales of the Short Form Survey (SF-36); depression (t(320) = 0.277, *p* = 0.782), anxiety (t(320) = − 0.668, *p* = 0.504) and stress (t(320) = 0.982, *p* = 0.327) scales of the Depression, Anxiety and Stress Scale (DASS-21); and life satisfaction (t(320) = − 0.605, *p* = 0.546) as measured by the Satisfaction With Life Scale. All baseline measures were within the normal range (Fig. 2).

**Table 2:** The Live More Project Intervention

Baseline characteristics	Intervention (n = 159)	Control (n = 162)
Age, mean (s.d.)	49.5 (14.3)	45.4 (14.2)
Sex, n (%)		
Men	48 (30.2)	43 (26.7)
Women	111 (69.8)	119 (73.3)
Ethnicity, n (%)		
White/Caucasian	128 (80.5)	135 (83.2)
Asian	10 (6.3)	6 (3.7)
Pacific Islander	5 (3.1)	11 (6.8)
Spanish/Hispanic/Latino	3 (1.9)	5 (3.1)
Indigenous	2 (1.3)	1 (0.6)
Black/African American	5 (3.1)	2 (1.2)
Other	6 (3.8)	2 (1.2%)
Education status, n (%)		
Primary/elementary	0 (0.0)	2 (1.2)
Secondary/high school	19 (11.9)	25 (15.5)
Tertiary/university undergraduate	75 (47.2)	72 (44.1)
Tertiary/university postgraduate	65 (40.9)	63 (39.1)

### Outcome measures

As illustrated in Fig. 2, the GLM analyses indicated that the intervention group experienced significantly greater improvements than the wait-list control group over time in all outcome measures (interactions between condition and time): mental health (F(319) = 7.326, *p* = 0.007), vitality (F(319) = 9.445, *p* = 0.002), depression (F(319) = 7.841, *p* = 0.005), anxiety (F(319) = 4.440, *p* = 0.360), stress (F(319) = 12.494, *p* =  < 0.001) and life satisfaction (F(319) = 8.731, *p* = 0.003). Post hoc analyses using independent samples t-tests indicated that the intervention group experienced significantly greater changes from Week 1 to Week 12 in all outcome measures: mental health (t(357) = − 3.958, *p* < 0.001, dz = 0.42), vitality (t(357) = − 4.279, *p* < 0.001, dz = 0.45), depression (t(357) = 3.195, *p* = 0.002, dz = 0.34), anxiety (t(357) = 4.325, *p* < 0.001, dz = 0.46), stress (t(357) = 3.508, *p* = 0.001, dz = 0.37) and life satisfaction (t(357) = − 4.702, *p* < 0.001, dz = 0.50). The improvements experienced by the intervention group remained significantly greater than the wait-list control at Week 24 compared to Week 1: mental health (t(319) = − 2.707, *p* = 0.007, dz = 0.30), vitality (t(319) = − 3.073, *p* = 0.002, dz = 0.34), depression (t(319) = 2.800, *p* = 0.005, dz = 0.31), anxiety (t(319) = 2.113, *p* = 0.035, dz = 0.24), stress (t(319) = 3.545, *p* < 0.001, dz = 0.40) and life satisfaction (t(319) = − 2.958, *p* = 0.003, dz = 0.33).

Within the intervention group, significant improvements were observed from Week 1 to 12 in all outcome measures: mental health (10%, t(167) = − 6.423), *p* < 0.001, dz = 0.50), and vitality (22%, t(167) = − 7.043, *p* < 0.001, dz = 0.54), depression (− 41%, t(167) = 6.189, *p* < 0.001, dz = 0.48), anxiety (− 38%, t(167) = 5.030, *p* < 0.001, dz = 0.39), stress (− 31%, t(167) = 6.702, *p* < 0.001, dz = 0.52), and life satisfaction (8%, t(167) = − 6.199, *p* < 0.001, dz = 0.48) as measured by SWLS (see Fig. 2 and Additional file 1). At Week 24, the changes in these measures from Week 1 remained significant for the intervention group: mental health (8%, t(158) = − 5.375, *p* < 0.001, dz = 0.43), vitality (19%, t(158) = − 5.997, *p* < 0.001, dz = 0.48), depression (− 40%, t(158) = 5.070, *p* < 0.001, dz = 0.40), anxiety (− 29%, t(158) = 4.211, *p* < 0.001, dz = 0.33), stress (− 26%, t(158) = 5.808, *p* < 0.001, dz = 0.46) and life satisfaction (8%, t(158) = − 5.875, *p* < 0.001, dz = 0.47).

In contrast, the wait-list control group experienced significant improvements from Week 1 to Week 12 only in mental health (2%, t(190) = − 2.210, *p* = 0.028, dz = 0.16) and stress (− 9%, t(190) = 2.092, *p* = 0.038, dz = 0.15), but not in vitality (4%, t(190) = − 1.710, *p* = 0.089, dz = 0.12), depression (17%, t(190) = 1.596, *p* = 0.112, dz = 0.12), anxiety (5%, t(190) = − 0.796, *p* = 0.427, dz = 0.05) or life satisfaction (0%, t(190) = 0.161, *p* = 0.872, dz = 0.14) (see Fig. 2 and Additional file 1). All measures for the wait-list control were similar at Week 24 to those at Week 1: mental health (2%, t(161) = − 1.382, *p* = 0.169, dz = 0.11), vitality (5%, t(161) = − 1.357, *p* = 0.177, dz = 0.11), depression (− 5%, t(161) = 0.598, *p* = 0.551, d = 0.05), anxiety (− 5%, t(161) = 0.444, *p* = 0.657, d = 0.03), stress (1%, t(161) = − 0.112, *p* = 0.991, dz = 0.01) and life satisfaction (1%, t(161) = − 0.365, *p* = 0.715, dz = 0.03).

At Week 24, the attrition rate was 24% for this study. There were no significant differences between the completors and dropouts in the following characteristics: age (*p* = 0.510), sex (*p* = 0.511), ethnicity (*p* = 0.081) and education (*p* = 0.307). Comparisons were made between those analysed (defined as those who undertook the intervention and all three assessments, n = 321) and the dropouts (defined as those who completed the baseline questionnaire but did not progress to completing the 12 weeks post-intervention questionnaire, n = 104). The data showed that the dropouts had significantly poorer scores for several of the mental health metrics including: mental health (75.4 ± 14.5 vs 59.1 ± 22.4, t(424) = 6.438, *p* < 0.001), depression (2.5 ± 2.6 vs 3.8 ± 4.1, t(424) = − 2.936, *p* = 0.004), anxiety (1.7 ± 1.9 vs 2.5 ± 2.7, t(424) = − 2.636, *p* = 0.010) and life satisfaction (23.8 ± 6.5 vs 22.2 ± 7.9, t(424) = 1.977, *p* = 0.49).

### Harms

There were no reported harms that arose through the trial. No harms were anticipated given that the cohort was a subclinical population and the strategies implemented were positive lifestyle and psychological behaviours.

## Discussion

To our knowledge, this is the first randomized controlled trial to examine the effectiveness of an interdisciplinary online intervention combining an array of strategies from Lifestyle Medicine and Positive Psychology on mental health and emotional wellness amongst a subclinical population. Hence, interventions such as those employed in this study may provide a response to the repeated calls for lifestyle interventions, psychological strategies, and education programs for the promotion of mental health and primary prevention of common mental health disorders [5, 8–11].

Targeting mental health promotion and prevention through interventions such as that used in the present study may provide a protective ‘buffer’ for individuals. For example, enhanced positive emotion may increase an individual’s ability to cope when faced with adversity [13], and hence increase their resilience. The broaden-and-build theory asserts that the experiences of positive emotions may strengthen an individual’s personal, social and psychological resources, sparking upward spirals in their emotional wellbeing [63]. In turn, this may have a moderating impact on stress, anxiety and depressive symptoms [64].

A unique aspect of the intervention used in this study was its multicomponent approach. To date, numerous studies have investigated the effectiveness of Positive Psychology techniques for improving mental health, including expressing gratitude, practising forgiveness, positive thinking and engaging in service activities [38, 39]; however, these strategies are often used in isolation. Similarly, while lifestyle factors such as diet [32] and exercise [28, 33, 65] are increasingly used as management and treatment modalities for mental illness, they are infrequently combined with psychological strategies.

It is notable that the effect sizes observed in this study are relatively greater than those reported in two meta-analyses of randomized controlled trials, however, the studies in the meta-analyses were of a significantly smaller sample size and predominantly employed single-modality psychological strategies for the primary prevention of mental health [14, 58–66]. Further research is required to understand the underlying mechanisms of the larger effect sizes observed in the present study and if the multicomponent approach resulted in a compounding effect. Encouragingly, within Positive Psychology greater attention is being given to combining an array of psychological strategies [67], however, other evidence-based lifestyle factors, such as physical activity and nutrition, are typically not integrated into these interventions.

Over the past decade, wellness interventions focusing on mental health have increasingly moved online, as compared to the conventional face-to-face mode of delivery [60–62]. Online interventions have been shown to be efficacious and may yield several advantages over face-to-face delivery, including cost-effectiveness, ease of delivery and scalability [68].

Interestingly, the outcomes observed in this study are generally comparable to those observed in a cohort study that utilised the same intervention delivered face-to-face as part of a mandatory class for tertiary students [47]. However, there are several confounders in comparing the results of the cohort study to the present study, namely the participants in the present study were self-selected, of a broader age range and were generally healthier. There is a need to further explore the relative outcomes of mental health interventions when delivered online as compared to face-to-face.

Notably, the attrition rate in this study (24% at Week 24) was relatively low compared to that typically reported in online interventions that do not involve the support of a health professional (often as high as 45%) [39, 69]. A number of factors may have contributed to the low attrition rates observed in this study including the experiential pedagogical framework of the intervention and its interactive components, which are known to be key contributors to creating engagement in online interventions [26].

The observation that those participants who dropped out had poorer mental health scores than those who completed the intervention is notable. There is evidence to suggest that poor mental health and negative emotional states are linked with poor lifestyle choices [70, 71]. Conversely, better mental health is associated with healthy lifestyle choices [72] and according to the “upward spiral” theory, positive affect can aid long-term adherence to positive health behaviours [73]. Indeed, there is a reciprocal relationship between mental health and physical health outcomes because of the behavioural implications of poor mental health [74]. The implication of this observation is that while individuals with poorer mental health might have the greatest need of, and stand to benefit most from mental health interventions, it may be more difficult for them to engage and take positive steps. Certainly, this presents as an important area for further investigation.

### Strengths and limitations

A strength of this study was that it involved participants from a broad age range (18 to 88 years) and geographically diverse regions. Another strength of the study is that the intervention used an e-learning management system that was not reliant upon follow-up support from a healthcare professional. This suggests that such interventions might represent a scalable, low-cost method for the promotion of mental health and emotional wellness on a population-level.

A limitation of this study is that the participants where self-selected and drawn from the same faith-based organization that funded the study, so they may have entered the intervention with a higher readiness for change than that of the general population, thus limiting the generalizability of the findings. Secondly, as commonly seen when participants self-select into a Positive Psychology intervention, the sex balance was skewed towards females, which may limit the generalizability of the intervention to male cohorts. Notably, studies of Positive Psychology interventions have not shown the mental health outcomes to be sex specific [14, 39, 41]. Thirdly, a limitation of this study was that it was registered retrospectively with the clinical trial registry, however, the full protocol for the study as documented in this publication was presented at an academic conference prior to data collection. Fourthly, as the study targeted mental health promotion, it does not inform the effectiveness of the intervention among a clinical population with a confirmed diagnosis. As discussed previously, this warrants further investigation given that a previous cohort study has indicated that individuals with lower levels of mental health reported the greatest benefits from the intervention [46].

### Implications

This study has important implications for research and practice. Firstly, an early intervention based on an interdisciplinary approach, incorporating Lifestyle Medicine and Positive Psychology strategies, can be used to achieve significant improvements in mental health and emotional wellness. Hence, Clinicians should consider promoting lifestyle medicine strategies and their benefits to improve mental health. Secondly, while the positive mental health outcomes are encouraging, future studies are required to investigate the relative contribution of the various evidenced-based strategies incorporated into the intervention, as well as who responds best to the intervention (i.e. age and sex effects). Thirdly, further research is required to explore the potential for a compounding effect, whereby combining a broad array of evidence-based approaches for improving mental health and emotional wellness may result in greater benefits than a single-modality approach. Lastly, online modes of delivery may present a low-cost and scalable opportunity for mental health promotion to improve mental health and emotional wellness.

## Conclusion

Online interdisciplinary interventions may be a cost-effective and scalable method of mental health promotion. There is a need for future studies to examine the impact of online interdisciplinary interventions on at-risk and clinical populations to assess their potential role in secondary and tertiary prevention.

## Supplementary Information

**Additional file 1: Supplementary Table 1**. Changes in the outcome measures across time.

**Additional file 2: Supplementary Table 2**. Correlations between the outcome measures.

## Data Availability

The data that support the findings of this study are available from Adventist Health through the Seventh-day Adventist Church (SPD) Ltd, but restrictions apply to the availability of these data, which were used under license for the current study, and so are not publicly available. Data are however available from the authors upon reasonable request and with permission of the Seventh-day Adventist Church (SPD) Ltd.

## Abbreviations

SF-36: Short Form Survey (36 items)
DASS-21: Depression, Anxiety and Stress Scale (21-Items)
SWLS: Satisfaction With Life Scale
TPB: Theory of planned behaviour
LETS: Learn, experience, think and share
GLM: General linear modelling
